# Focal Myopericarditis Presenting as Acute ST-Elevation Myocardial Infarction

**Published:** 2018-08-30

**Authors:** Jeet J. Mehta, Mohinder R. Vindhyal, Venkata S. Boppana, Assem Farhat

**Affiliations:** 1 University of Kansas School of Medicine-Wichita, Internal Medicine/Pediatrics Residency Program, Wichita, KS; 2 Heartland Cardiology, Wichita, KS

**Keywords:** myocarditis, pericarditis, ST elevation myocardial infarction, myocardial ischemia

## INTRODUCTION

Myopericarditis is an inflammation of the myocardial and the pericardial layers of the heart.^1^ Acute myopericarditis commonly is caused by viral illness, however less commonly, it may be caused by non-infectious etiologies. Myocarditis, which is an inflammation of the myocardium, can be divided into acute, sub-acute, or chronic. Based on the histology, these can be classified as active or borderline myocarditis by the Dallas criteria for interpretation of endomyocardial biopsy. The disease may either affect a focal part of the myocardium or have diffuse involvement.

The incidence of myocarditis and pericarditis is 22 – 27.7/100,000 and the incidence of myopericarditis is even less.^2^ Myocarditis is reported to be the reason for 0.5% to 3.5% of the heart failure hospitalizations all over the world.^3^ While myocarditis is a well-known presentation, focal myopericarditis is rare. Focal myopericarditis can mimic an acute ST-elevation myocardial infarction, which often requires prompt treatment with invasive angiography. This identical presentation makes diagnosis of focal myopericarditis difficult, and thus, clinical suspicion is essential for diagnosis. With growing population presenting as MI from coronary plaque ruptures, the diagnosis of focal myopericarditis remains challenging. Its clinical presentation may vary from subtle to life-threatening to even death.

We present a Caucasian female with chest pain and ST-elevation on electrocardiogram (ECG) who was found to have focal myopericarditis.

## CASE REPORT

An 18-year-old previously healthy female presented to the emergency room for crushing chest pain. She woke up at 0800 with mid-sternal chest pain radiating to her back and bilateral arms with associated symptoms of shortness of air at rest. She denied cough, rhinorrhea, nasal congestion, nausea, vomiting, diarrhea, or diaphoresis. She reported no change in quality of pain with position or inspiration. She did not have any past surgical history or history of premature coronary artery disease in her family. The patient smoked 1 – 2 cigarettes per day, but denied illicit drug or alcohol abuse.

**Figure 1:**
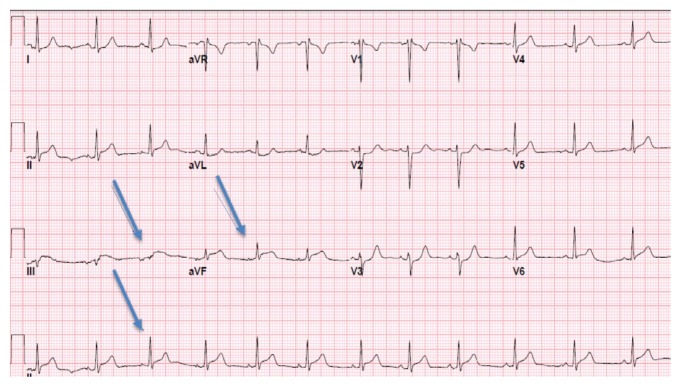
Admission ECG showing ST-segment elevation in inferior leads II, III, and aVF (arrows).

Physical examination in the emergency room was notable for regular heart rhythm without murmurs, gallops, or rubs. She was afebrile with a heart rate of 74 bpm, respiratory rate of 12, blood pressure of 137/93 mmHg, and oxygen saturation was 99% on room air. She had no peripheral edema or jugular venous distention. Her lungs were clear to auscultation. An electrocardiogram (ECG) on presentation showed ST-elevation in the inferior wall distribution (Leads II, III, and aVF) with non-specific PR interval changes (Figure 1). Troponin I was 0.06 ng/mL on admission (reference normal <0.07ng/mL), which peaked to 22.88 ng/mL at 12 hours after admission. CT-angiography of the chest ruled out aortic dissection and pulmonary embolism. Echocardiogram at bedside revealed inferior wall hypokinesis, which was concerning for a plaque rupture, thus, she was taken for cardiac catheterization while the troponin was trending up (from 0.06 to 1.06 ng/mL four hours after admission).

**Figure 2:**
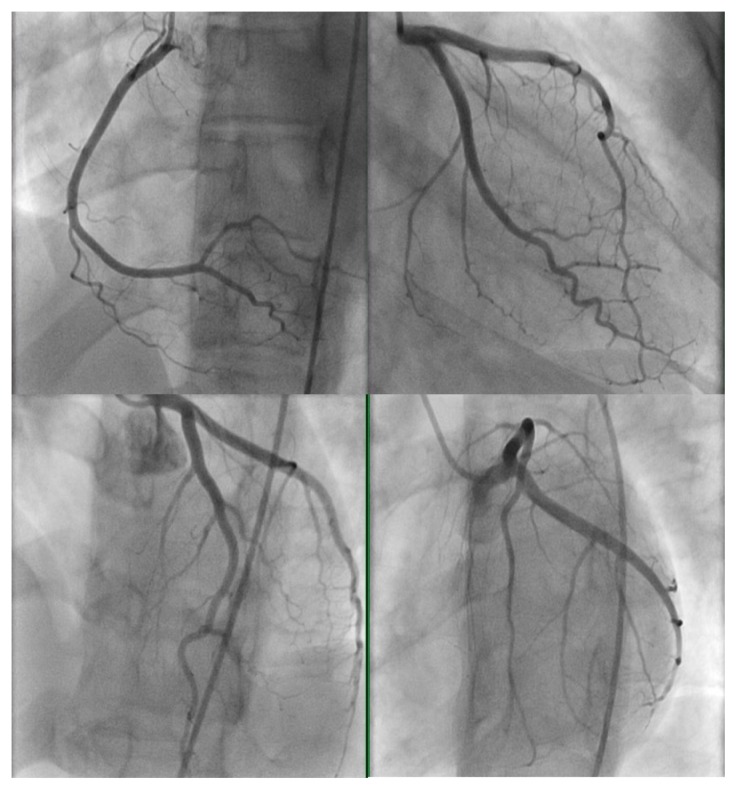
Angiography of the left heart vessels shows patent right (top left image) and left coronary (top right and lower images) arteries and branches.

Cardiac catheterization revealed patent coronary arteries with hypokinesis of the inferior wall on left ventriculogram (Figure 2). Echocardiogram showed left ventricular ejection fraction of 55 – 60% with mild degree of hypokinesis in the inferior wall. Complete blood count, renal function panel, thyroid function studies, erythrocyte sedimentation rate, C-reactive protein, and urine drug screens were normal. Fasting lipid panel showed elevated total cholesterol at 213 mg/dL and low density lipoprotein (LDL) at 136 mg/dL with a normal high density lipoprotein (HDL) at 66 mg/dL. Because of a negative heart catheterization with ST segment elevation on ECG and troponin elevation, coronary vasospasm and myopericarditis were high among the differential diagnosis. Troponin I trended down after the 12-hour peak, and the patient was stable with chest pain resolved. She was discharged after 24 hours to have a close outpatient follow-up for a cardiac MRI (CMR). With the exception of nonsteroidal anti-inflammatories as needed, no new medications, including anticoagulants, were prescribed at the time of discharge.

**Figure 3:**
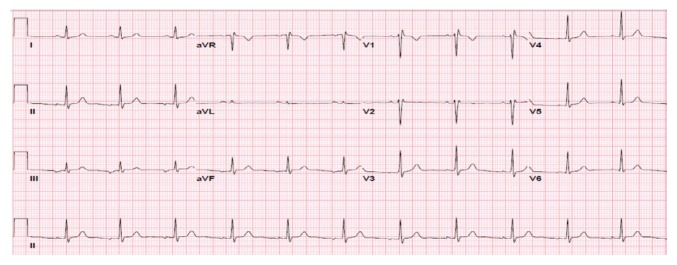
A 2-week follow-up ECG showing resolution of the ST-segment elevation.

**Figure 4:**
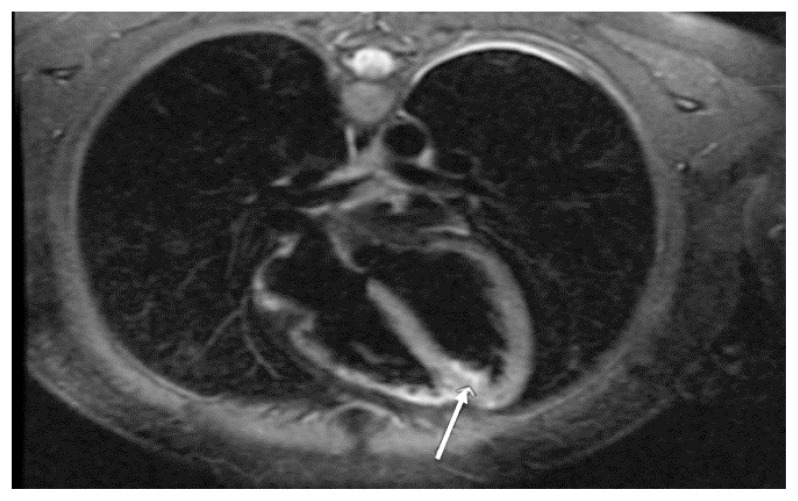
CMR showed epicardial and transmural delayed enhancement, suggestive of focal myopericarditis (arrow).

At two-week follow-up, the patient was symptom-free with a normal ECG (Figure 3). To support the clinical diagnosis of myopericarditis and rule out other possible causes of non-ischemic cardiomyopathy, she underwent a cardiac magnetic resonance imaging (CMR) as an outpatient, which revealed hypokinesis of the lateral and inferior wall of the left ventricle apex with epicardial and transmural delayed enhancement, suggestive of focal myopericarditis (Figure 4).

## DISCUSSION

Given the patient’s age, drug abuse causing vasospasm, aortic dissection, and thromboembolic causes were high on the differential diagnoses, but these were ruled out by history, vital signs, and administration of sublingual nitroglycerin. Myocarditis and pericarditis were low on the differential as they typically present with diffuse ST segment changes on ECG. Clinical presentation is the key in this particular diagnosis; however in our patient, she denied having any prodromal viral or gastrointestinal illness which made it more difficult. Of note, our patient remained afebrile during the entirety of her hospitalization. Several factors can trigger coronary vasospasm, such as use of cocaine, amphetamine, marijuana, chemotherapy drugs, over-the-counter medications, and antibiotics. Our patient did not have exposure to any of these.

Coronary artery spasm is a multifactorial disease with underlying mechanism that is still poorly understood.^4^ Typically, coronary artery spasm will reveal stenosis during cardiac catheterization during both systole and diastole and responds to calcium channel blockers and nitroglycerin. Myopericarditis typically follows history of fever. A history of upper respiratory infection symptoms often helps, but its absence does not rule out the disease. Ultimately, to differentiate the two, diagnostic tools such as enhanced imaging techniques like CMR and/or invasive coronary angiography come into play to make the correct diagnosis.

ST-elevation myocardial infarction is the most important differential diagnosis of focal myopericarditis among others.^5^ Myopericarditis shows a characteristic pattern of contrast enhancement on CMR, which originates primarily from the epicardium, sparing the subendocardial layer. Myopericarditis need not always have elevated ESR and CRP as they are only positive in 60% of the cases.^6^ Indications for endomyocardial biopsy (EMB) include new-onset heart failure of less than two weeks duration associated with hemodynamic compromise which is unexplained, new-onset heart failure of two weeks to three months duration associated with a dilated left ventricle, new ventricular arrhythmias with no certain explanation, Mobitz type II second-degree atrioventricular (AV) block, third-degree atrioventricular (AV) block, or refractory heart failure.^7^ Biopsy is indicated in these instances to evaluate for possible rare diagnoses such as giant cell myocarditis (where early diagnosis prompts potential preparation for transplantation), lymphocytic myocarditis, sarcoidosis (response to restrictive cardiomyopathy from steroids which are not otherwise used for routine heart failure treatment), dilated cardiomyopathy due to eospinophilia (high-dose steroid responsive), amyloidosis, and hemochromatosis. These different etiologies have unconventional heart failure treatment and present acutely, thus EMB may help in this situation. Dallas Criteria (histologic criteria) have an overall low diagnostic yield according to one case series of 1,230 patients.^8^

Diagnosis of myopericarditis is empiric and made on clinical presentation. ECG changes, elevated cardiac enzymes, lack of epicardial coronary artery disease, and abnormal CMR support the diagnosis.^9^ Myopericarditis shows a characteristic pattern of contrast enhancement on CMR, which originates from the epicardium, sparing the subendocardial layer.^10^ Positron emission tomography (PET) also can be used to assess for occult inflammation.^11^ Indications for endomyocardial biopsy exist, but the sensitivity and specificity of EMB are approximately 60% and 80%, respectively, and autopsy is the gold standard.^12^ A lower sensitivity of 35% has been noted when a clinical and functional gold standard is used.^13^ Treatment is supportive for myopericarditis and case-dependent. Clinicians must have a high clinical suspicion for the diagnosis of myopericarditis with elevated cardiac enzymes and ST elevation in absence of coronary artery disease.
